# Chemokine-Like Factor 1 (CKLF-1) is Overexpressed in Keloid Patients: A Potential Indicating Factor for Keloid-Predisposed Individuals

**DOI:** 10.1097/MD.0000000000003082

**Published:** 2016-03-18

**Authors:** Mingzi Zhang, Ying Xu, Yifang Liu, Yingying Cheng, Pengxiang Zhao, Hao Liu, Youbin Wang, Xuemei Ma

**Affiliations:** From the Department of Plastic Surgery (MZ, HL, YW), Peking Union Medical College Hospital; Department of General Surgery (YX), Youan Hospital Capital Medical University; College of Life Science and Bioengineering (YL, PZ, XM), Beijing University of Technology; and Peking University Center for Human Disease Genomics (YC), Peking University, Beijing, China.

## Abstract

Chemokine-like factor 1 (CKLF-1) is a novel cytokine which have a crucial role in immune and inflammatory responses. In this study, the expression level of CKLF-1 was measured to assess the difference between keloid patients and people without keloid.

Fifty samples were taken from 30 patients: 10 keloid patients; 10 scar patients; and 10 patients without obvious scarring. Patients were randomly selected from the hospitalized patients of Peking Union Medical College Hospital from September 2013 to July 2015. Five groups of samples were established: keloid samples from keloid patients (K); normal skin samples from keloid patients (KS); scar samples from scar patients (C); normal skin samples from scar patients (CS); and normal skin samples from patients without obvious scarring (S). Hematoxylin and eosin (H&E) staining was used to observe morphological changes. CKLF-1, IL-6, IL-8, IL-18, and TGF-β were detected by immunohistochemical and western blot technology. The expression of CKLF-1's mRNA was also measured by the real-time quantitative polymerase chain reaction (RT-qPCR).

Compared to the K group, the other 4 groups presented significantly less inflammatory infiltration and lower expression levels of CKLF-1, IL-6, IL-8, IL-18, and TGF-β. Among the 3 normal skin groups, the expression level of CKLF-1 was significantly higher in the KS group than in the CS or S group. The mRNA expression was also obvious in the K and KS groups.

CKLF-1 and other inflammatory factors were overexpressed in the samples from keloid patients, indicating that the formation of keloid may be related to inflammation and that CKLF-1 may play an important role in this process.

## INTRODUCTION

Keloid, considered to be a result of abnormal wound healing, is defined as excessive scar tissue formation extending beyond the area of the original skin injury and occurring in predisposed individuals.^1^ The main symptoms of keloid patients are pain, itching, functional limitation, and disfigurement, which may cause psychological distress and seriously affect patients’ quality of life.^2^ The mechanism of keloid formation is still unclear.

In keloid tissue, fibronectin and type I procollagen is overexpressed, and the degradation of procollagen polypeptides is reduced.^3^ In addition, the increased expression of growth factors such as transforming growth factor-β (TGF-β), platelet-derived growth factor-α (PDGF-α), and vascular endothelial growth factor (VEGF) has been detected.^1^ Altered responses to apoptotic signals and extracellular matrix remodeling were demonstrated by some studies.^1^ Inflammatory factors have been demonstrated to potentially have some relationship with keloid formation, especially interleukin-6 (IL-6), IL-8, and IL-18. In Arno's study, IL-6, IL-8, and TGF-β protein expression was found to be enhanced on keloid fibroblasts in vitro.^4^ Do et al demonstrated that IL-18 expression was elevated in keloid tissue, which plays an important role in keloid pathogenesis via epithelial-mesenchymal interactions.^5^

Chemokine-like factor 1 (CKLF-1) is a novel human cytokine isolated from PHA-stimulated U937 cells.^6^ The open reading frame of CKLF-1 cDNA encodes a highly basic and hydrophobic polypeptide of 99 residues with a molecular mass of 10.9 kD.^7^ CKLF-1 is widely expressed in human tissues. It has been reported that the CKLF-1 expression is increased in inflammatory and autoimmune diseases such as rheumatoid arthritis and asthma.^6,8–9^ CKLF-1 was also studied in the context of dermatitis. In Yang's study, they found that the protein and mRNA expression of CKLF-1, IgE, IL-4, IL-5, and IL-13 and the eotaxin level was significantly higher in atopic dermatitis patients than in healthy controls.^10^ In this study, we measured and assessed the expression level of CKLF-1 in keloid and other scar and skin tissue, and we speculated on the role and significance of CKLF-1 in keloid occurrence and development.

## METHODS

### Patients, Grouping, and Sample Treatment

This clinical study protocol was reviewed and approved by the Bioethical Committee of Peking Union Medical College Hospital. Informed consent was provided by all of the patients. We randomly selected 30 patients from the department of plastic surgery, Peking Union Medical College Hospital: 10 keloid patients (5 women and 5 men), 10 scar patients (4 women and 6 men), and 10 patients (5 women and 5 men) without any obvious scarring, with ages range from 18 to 48 (average age: 32.36). There is no significant difference of age, sex, and site between each group (*P *>* *0.05). The keloid and scar (caused by trauma) diagnoses were made and confirmed by a plastic surgeon and pathological examination. Before the study, none of the patients had any systematic disorder or were taking any drugs that might affect the results of this study.

**FIGURE 1:**
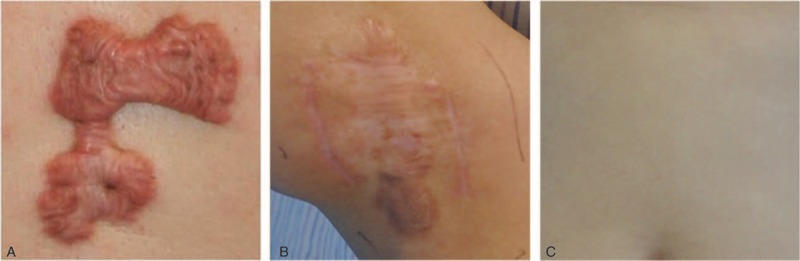
Sampling conditions of the 3 different groups of patients: (A) keloid tissue from keloid patients; (B) normal scar tissue from scar patients; (C) normal skin tissue from patients without obvious scarring.

Two specimens (1 keloid or scar sample and 1 normal skin sample) were taken from each of the 10 keloid patients and 10 scar patients. One normal skin sample was taken from each of the other 10 patients (Figure 1). In this study, the 50 samples were naturally divided into 5 groups: keloid samples from keloid patients (K); normal skin samples from keloid patients (KS); scar samples from normal scar patients (C); normal skin samples from scar patients (CS); and normal skin samples from patients without obvious scarring (S). Each specimen was cut into 3 parts: 1 was put into 10 % formalin solution for paraffin embedding to perform immunohistochemical studies and hematoxylin and eosin (H&E) staining; 1 was stored in liquid nitrogen for western blot analysis; and the last one was immediately immersed in RNA extraction solution and used for the real-time quantitative polymerase chain reaction (RT-qPCR).

### H&E Staining and Immunohistochemical Study

Specimens were fixed in a 10% formalin solution for 48 hours then embedded in paraffin, sectioned, and mounted on a slide. The tissue slides were stained with H&E for histological examination. For immunohistochemical staining, a standard indirect immunohistochemical method was used. Paraffin-embedded sections were routinely dewaxed and rehydrated and then incubated for 10 minutes with 3% H_2_O_2_ to block endogenous catalase. Antigen retrieval was performed by heating the unstained slides in citrate buffer to 95°C for 15 minutes. Incubation with normal goat serum at 37°C for 30 minutes blocked nonspecific staining. Sections were placed in a humidified chamber at 37°C for 2 hours with anti-CKLF-1 (1:100, Peking University Center for Human Disease Genomics, Peking University, Beijing, China), anti-IL-6 (1:200, Abcam, Cambridge, Britain), anti-IL-8 (1:200, Abcam, Cambridge, Britain), anti-IL-18 (1:200, Abcam, Cambridge, Britain), or anti-TGF-β (1:200, Abcam, Cambridge, Britain) antibody. Horseradish peroxidase-conjugated secondary antibody (ZSGB-BIO, Beijing, China) was used to mark the primary antibody. Then, the samples were flushed with phosphate buffered saline (PBS), stained with 3, 3′-diaminobenzidine (DAB) and afterwards counterstained with hematoxylin. A brown color implied the presence of antibody bound to antigen and was detected by light microscopy with a computer-controlled digital camera and imaging software. The positive condition was measured by estimating the color: brown staining indicated a positive expression area, and the shade of the color represents the expression level of the target protein.

### Western Blot Analysis

Samples of 50 mg were weighed, and protein was extracted with a Cell lysis kit (Bio-Rad laboratories, Hercules, CA). Samples were incubated on ice for 10 minutes in buffer (246 μL Lysis Buffer, 1.25 μL phosphatase inhibitor, 0.25 μL protease inhibitor, 2.5 μL PMSF) and then centrifuged. For western blotting, equal amounts of supernatant protein (60 μg) were separated by 10 % SDS-PAGE and transferred to nitrocellulose membranes for immunoblotting. The membrane was blocked with blocking buffer (Li-cor, Lincoln, NE) for 2 hours and then incubated with either anti-CKLF-1 (1:100, Peking University Center for Human Disease Genomics, Peking University, Beijing, China), anti-IL-6 (1:500, Abcam, Cambridge, Britain), anti-IL-8 (1:500, Abcam, Cambridge, Britain), anti-IL-18 (1:500, Abcam, Cambridge, Britain), anti-TGF-β (1:500, Abcam, Cambridge, Britain), or anti-β-actin (1:2000, Santa Cruz Biotechnology, Dallas, TX) antibody for 12 hours at 4°C. The membranes were incubated with secondary antibodies (Li-cor, Lincoln, NE) at 1:10,000 dilution in the dark for 1 hour at room temperature and then detected with a double color infrared laser imaging system (Odyssey, Li-cor, Lincoln, NE).

### RNA Isolation and RT-qPCR Technology

Samples of 30 mg were weighed, and total RNA was extracted with the RNeasy Fibrous Tissue Mini Kit (Qiagen, Düsseldorf, Germany) according to the manufacturer's instructions. The concentration of the extracted RNA was determined using a UV spectrophotometer (Thermo, Waltham, MA) and the integrity of the RNA was visualized by 1 % agarosegel electrophoresis. Reverse transcription of 1 μg total RNA for cDNA synthesis was performed using the ProtoScript M-MuLV First Strand cDNA Synthesis Kit (New England Biolabs, Ipswich, MA) and an anchored oligo-d(T) primer [d(T)23VN]. The process of amplification and quantification were performed by using the real-time qPCR System (Agilent, Santa Clara, CA). The primers for human CKLF-1 and β-actin were as follows: CKLF-1 forward, 5′-TCGCTTCGCAGAACCTACTCA-3′, reverse, 5′-TATTTTCGGCTGCACGTTATCC-3′;^8^ β-actin forward, 5′-GAAGGAAGGCTGGAAGAGTG-3′, reverse, 5′-GGAAATCGTGCGTGACATTA-3′. The expression of target genes was normalized by using the β-actin gene as an internal housekeeping control. Real-time qPCR cycle parameters included UDG pretreatment at 50°C for 2 minutes, initial denaturation at 95°C for 10 minutes followed by 40 cycles involving denaturation at 95°C for 30 seconds, annealing at 56°C for 30 seconds, and extension at 72°C for 30 seconds.

### Statistical Analysis

All the data presented in this study are mean ± standard deviation. Statistical significance was determined via 1-way analysis of variance (ANOVA) followed by the LSD-t test. The correlation analysis among factors was determined via Pearson correlation test. Statistical significance was set at *P* < 0.05. All analyses were conducted using SPSS 18.0.

## RESULTS

### Histological Analysis

**FIGURE 2:**
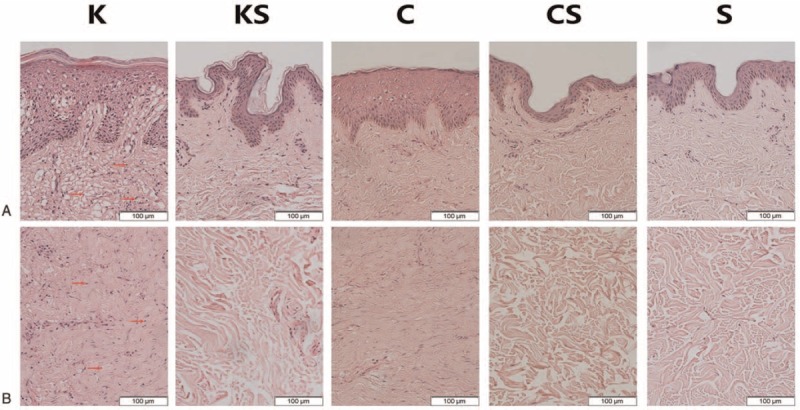
The results of H&E staining of epidermis (A) and dermis (B) in all groups. The number of infiltrated cells (red arrow) is much lower in the KS group, C group, CS group, and S group than in the K group (images: 200×). The epidermis of keloid and scar tissue is thicker than that of normal skin tissue. In keloid tissues, a large amount of fibroblasts with more cytoplasm and clear nucleoli are observed in the papillary layer and reticular layer of the dermis. Irregular, thick and extremely compact collagen fibrils appear disordered. By contrast, the nucleolus of normal skin tissue fibroblasts is small and there is less cytoplasm. In scar tissues, the collagen fibrils are relatively compact. Fibroblasts manifest more cytoplasm and a small nucleolus. C group = scar samples from normal scar patients, CS group = normal skin samples from scar patients, H&E = hematoxylin and eosin, K group = keloid samples from keloid patients, KS group = normal skin samples from keloid patients, S group = normal skin samples from patients without obvious scarring.

H&E stained tissue slices were used to confirm the pathologic examination and assess the inflammatory condition (Figure 2). Different pathologic morphological structures could be observed in each group. Inflammatory infiltration could be barely observed in the KS group, C group, CS group, and S group, but more inflammatory cells were found in the dermal layer of the skin tissue slices from the K group. According to the results above, there is active inflammatory infiltration in keloid tissues.

### CKLF-1, IL-6, IL-8, IL-18, and TGF-β Immunohistochemical Studies

**FIGURE 3:**
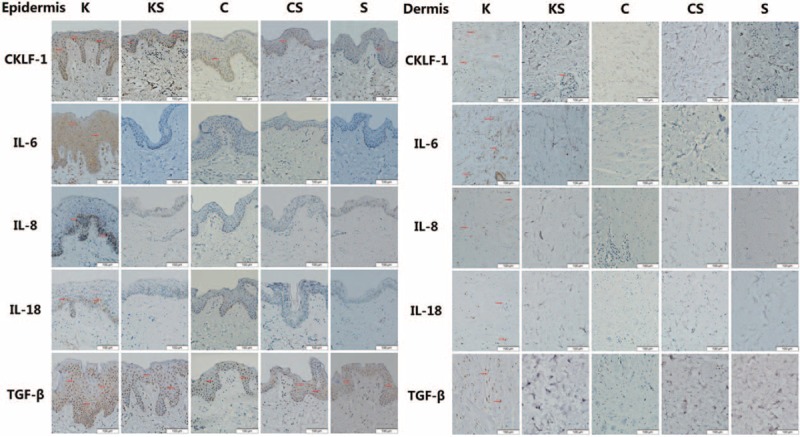
Representative micrographs (400×) of skin epidermal and dermal tissue immunohistochemistry for CKLF-1, IL-6, IL-8, IL-18, and TGF-β in all 5 groups are presented above. Brown staining indicates positive expression areas, and the shade of the color represents the expression level of the target protein. CKLF-1 is expressed in all types of skin tissue, but in the K group and KS group, the CKLF-1 expression level were (+++) and (++) compared to the other 3 groups. The levels of IL-6, IL-8, IL-18, and TGF-β are much higher in the K group than in the other groups.CKLF-1 = chemokine-like factor 1, IL = interleukin, TGF-β = transforming growth factor-β.

Immunohistochemical studies reflect the expression of target proteins such as CKLF-1, IL-6, IL-8, IL-18, and TGF-β. The results showed that the expression of epidermal and dermal CKLF-1, IL-6, IL-8, IL-18, and TGF-β was more obvious in the K group than in the KS, C, CS, or S group (Figure 3). CKLF-1 was expressed in all types of skin tissue, but in the K group and KS group, the CKLF-1 expression level was (+++) and (++) compared with (+) in the other 3 groups. TGF-β was also expressed in all groups, but the TGF-β level was (++) in the K group compared with (+) in the other 4 groups. As for IL-6, IL-8, and IL-18, their expression level was higher in the K group as well, whereas the other 4 groups showed low-level expression. There were 3 slices in each sample. In the K group, 83.33% slices of CKLF-1 showed moderate to strong intensity whereas it is 50.00% in the KS group, 10.00–18.33% in the other groups. As for IL-6, IL-8, IL-18, and TGF-β in the K group, 91.67 % (IL-6), 23.33 % (IL-8), 1.67 % (IL-18), and 63.33 % (TGF-β) slices showed moderate to strong intensity.

Percentage of immunohistochemical staining positive staining cells is shown in Table 1. The K group presents the highest positive cell percentage of all factors. There is significant difference of CKLF-1-positive cells between the KS group and S group both in epidermis and dermis.

**TABLE 1:**
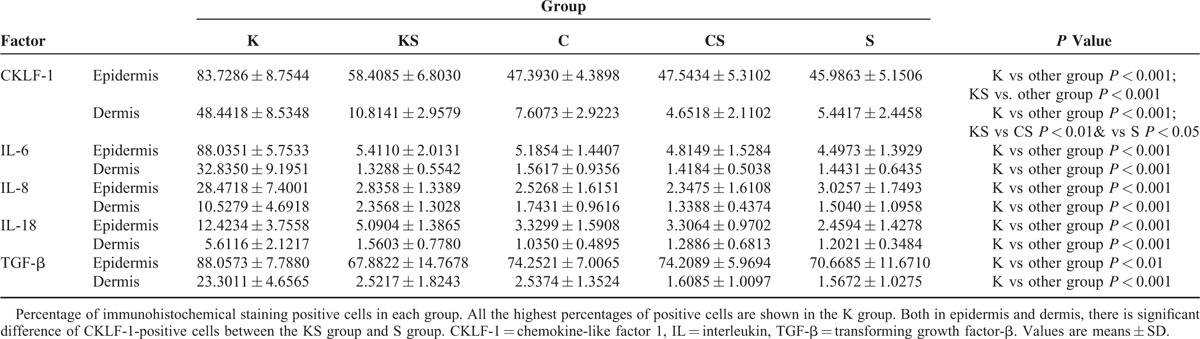
Percentage of Positive Cells in Immunohistochemical Staining Analysis of Each Group

### Expression of CKLF-1, IL-6, IL-8, IL-18, and TGF-β Protein

**FIGURE 4:**
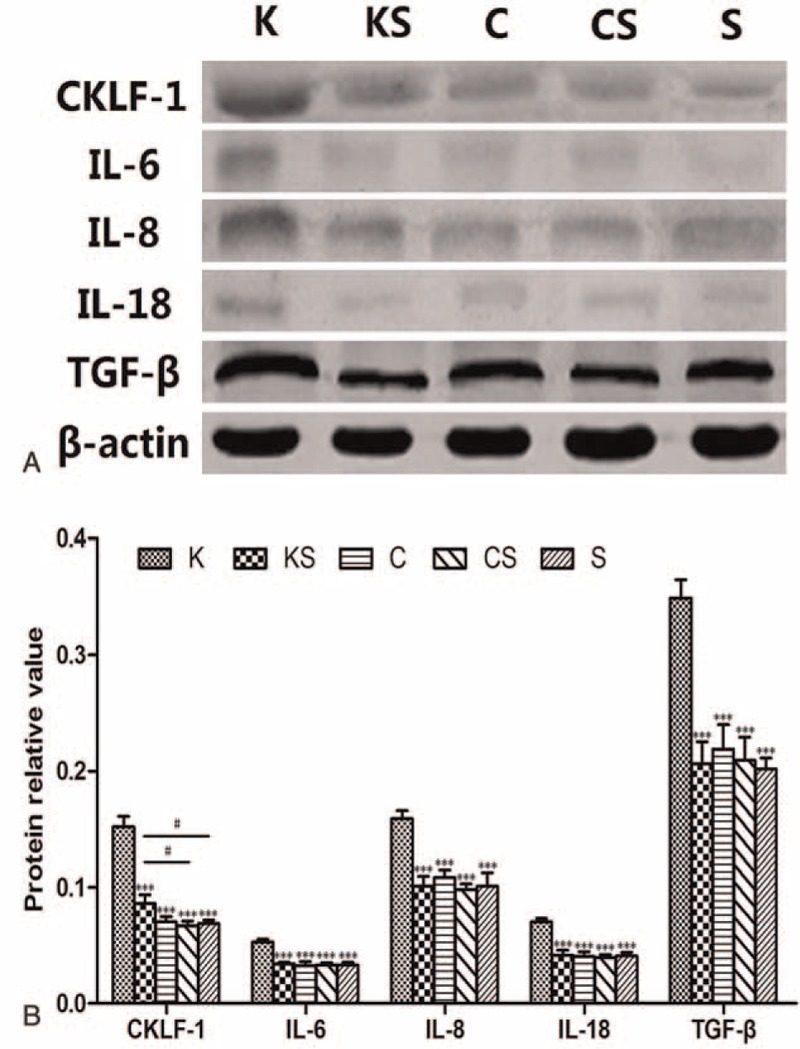
Protein expression of CKLF-1, IL-6, IL-8, IL-18, and TGF-β. (A) Representative images of western blots for CKLF-1, IL-6, IL-8, IL-18, and TGF-β are shown. (B) Densitometry analysis of CKLF-1, IL-6, IL-8, IL-18, and TGF-β protein levels, the results are consistent with the immunohistochemistry. Values are means ± SD (n = 10, ^∗∗∗^*P* < 0.001 vs the K group, ^#^*P* < 0.05). CKLF-1 = chemokine-like factor 1, IL = interleukin, TGF-β = transforming growth factor-β.

The protein expression of CKLF-1, IL-6, IL-8, IL-18, and TGF-β in skin tissues of the 5 groups was visualized by western blot (Figure 4A and Table 2). In group K, the expression of CKLF-1 was much higher than that in the other 4 groups. In addition, there were significant differences in CKLF-1 expression between the KS and CS groups and the KS and S groups (Figure 4B).

**TABLE 2:**
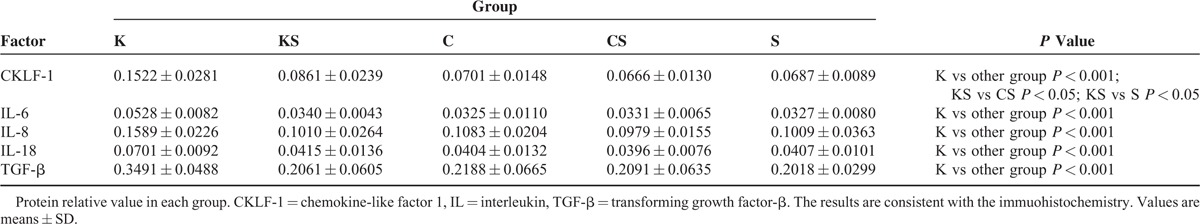
Protein Relative Value of CKLF-1, IL-6, IL-8, IL-18, and TGF-β in Each Group

The expression of IL-6, IL-8, IL-18, and TGF-β was much higher in the K group than in the other 4 groups (Figure 4B). Above all, CKLF-1, IL-6, IL-8, IL-18, and TGF-β were overexpressed in the K group. Among the 3 normal skin groups, CKLF-1 expression was higher in the KS group with a significant difference compared with the CS and S groups.

The correlation results between the expression of CKLF-1 and IL-6, IL-8, IL-18, TGF-β are shown in Table 3. There are correlations between the expression of CKLF-1 and other inflammatory factors, which are significant.

**TABLE 3:**
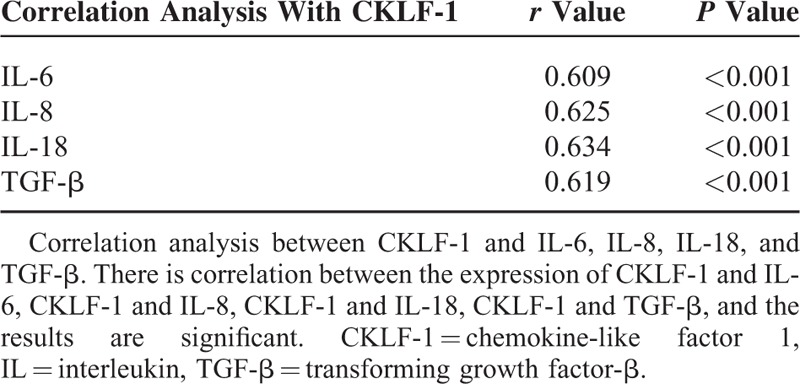
Correlation Analysis Between the Expression of CKLF-1 and Other Inflammatory Factors

### CKLF-1 Modulation at the mRNA Level

**FIGURE 5:**
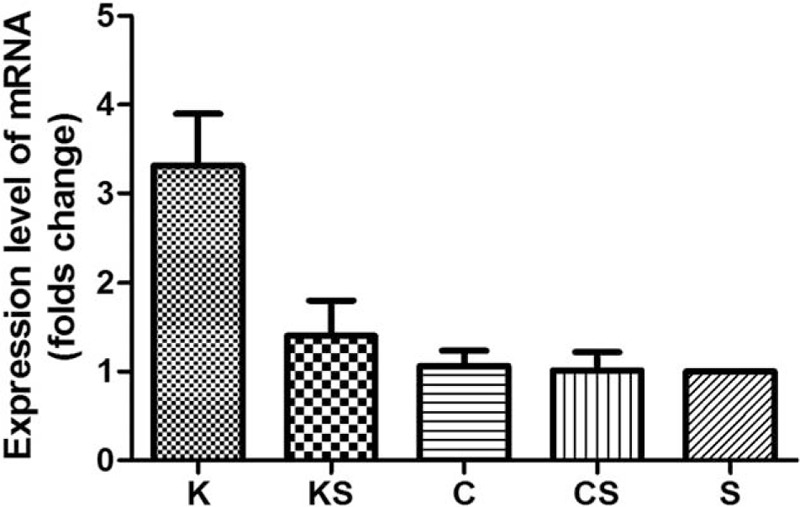
mRNA expression level of CKLF-1 in the 5 skin sample groups is analyzed. Compared to the S group, CKLF-1 mRNA is upregulated in the K and KS groups. The mRNA expression in the C group and CS group is almost at the same level as that of the S group. CKLF-1 = chemokine-like factor 1.

The CKLF-1 mRNA expression level in the K group was 3.3125 ± 1.8529 times higher than that in the S group, and in the KS group it was 1.4035 ± 1.2641 times higher. The mRNA level of CKLF-1 in the C group (1.0641 ± 0.5379 times) and CS group (1.0147 ± 0.6398 times) was almost at the same level as that of the S group (Figure 5).

## DISCUSSION

Keloids usually occur as a result of pathological wound healing after trauma and inflammation.^11–13^ The scars are associated with physical discomfort, functional limitation, and significant psychological morbidity because of their disfiguring appearance.^14–15^ In Lee's study,^16^ 46% of patients noted keloid-associated pain and 86% mentioned pruritus. Keloids most commonly occur in darker-skinned individuals, occurring 15 times more often than among Caucasians, suggesting a genetic factor. Many cytokines and growth factors are altered during the healing process, such as TGF-β, PDGF-α, and VEGF.^1^ Some genes have also been found to be related to keloid formation. Compared with normal fibroblasts, keloid fibroblasts have high levels of p53 and Stat-3 expression.^17,18^ In other genetic research, various human leukocyte antigen (HLA) types,^19–22^ including HLA-DR5, HLA-DQ23, HLA-DQA1, and HLA-DQB1, have been found to correlate with keloid phenotype in a Caucasian and Chinese Han population, suggesting that the immune reaction might play a role in keloid formation. In clinical work, combined approaches are used as effective treatment to decrease the high rate of recurrence after surgical excision.

Three stages comprise normal wound healing: the inflammatory stage,^23^ the proliferative/granulation stage,^24^ and the maturation/remodeling stage.^25^ After skin injury, inflammation first occurs in local tissue. Interleukins, interferon, and growth factors are common inflammatory factors, among which interleukins and growth factors play a paramount role in initiating inflammation.^26^ IL-6, IL-8, and IL-18 participate in inflammation. IL-6 can promote T-cell proliferation and presents some synergistic effects with IL-1, such as leading to fever reaction and increasing acute-phase proteins (APPs).^27^ IL-8, previously called aneutrophil-activating factor, aims to attract and activate neutrophils, which are the main participants during inflammation.^28^ IL-18 belongs to the proinflammatory cytokines, regulating inflammatory cell proliferation, and their secretion function, which suggests an important role in early inflammation.^29^ TGF-β has been reported to affect tissue repair and inflammation. TGF-β can improve the release of IL-6 and promote the proliferation of thymic cells with IL-6.^30^

Cytokines are small proteins that play an essential role in the immune reaction and inflammatory responses. CKLF-1 was first reported in Han's study in 2001. The full-length cDNA of CKLF-1 is 530-bp long and has a single open reading frame encoding 99-amino-acid residues.^31^ In addition, the study concluded that CKLF-1 might have an important role in inflammation and demonstrated its potential chemotactic activity. Recently, the biological effects of CKLF-1 have been explored more deeply. Li's study^32^ indicated that CKLF-1 could activate NF-κB and the large number of genes regulated by NF-κB, contributing to asthma. In skin disease, Yang's study^10^ also concluded that CKLF-1 is overexpressed in atopic dermatitis (AD) patients, and it suggests CKLF-1 may be a new target for the treatment of patients with AD.

In our study, 5 groups were established, containing 50 samples from 30 patients. The histological analysis performed by H&E staining showed more infiltrated cells in the K group, which implies there was active inflammation in keloid tissues. The existing data of former studies suggest that IL-6, a key cytokine with pro-inflammatory function, mediated inflammation is a key player and may be considered as a common causative factor for keloid development.^33,34^ It has been accepted that the formation of keloid follows with the change of inflammatory factors, such as IL-6, IL-8, IL-18, and TGF-β. The protein expression of IL-6, IL-8, IL-18, and TGF-β was higher in the K group than in the other 4 groups, which is consistent with the results of former studies.^4,33–36^ The high-level expression of IL-6, IL-8, and IL-18 indicates the degree of inflammation present in keloid tissues, which present with more inflammatory cell infiltration. TGF-β stimulates fibroblast production and the deposition of collagen and extracellular matrix (ECM) factors to promote the wound-healing process.^36^ In addition, keloid tissues showed high levels of CKLF-1 protein and upregulated mRNA expression. Li's study^32^ and Yang's study^10^ also concluded that CKLF-1 may be a new target in inflammatory diseases. In our study, the correlation between CKLF-1 and inflammatory factors has been analyzed, and our conclusion demonstrates the existence of this correlation. However, samples in the KS group also showed a higher level of CKLF-1 expression among the normal skin groups, which means the basal expression of CKLF-1 was increased in keloid individuals over that in other people without keloid.

According to the results above, both the protein and mRNA expression of CKLF-1 were higher in keloid tissues than in normal skin tissues or scar tissues. However, another interesting result occurred in our study: between the KS group and the other 2 normal skin groups, there was a significant difference in CKLF-1 expression; however, no significant difference in IL-6, IL-8, IL-18, or TGF-β expression was found. To analyze this phenomenon, we considered the following possibility: first, according to the expression results of CKLF-1 and H&E staining, CKLF-1 protein levels were higher in the K group with active inflammation. By contrast, the other groups showed light inflammatory cell infiltration. Moreover, keloid patients usually have a history of skin damage.^24^ Therefore, CKLF-1 might have the effect of enhancing the inflammatory reaction or extending the inflammation time after particular stimuli such as skin injury. However, normal skin tissue in the absence of such stimulation may not activate the CKLF-1 expression that leads to the low level of inflammatory factors. Second, the expression results of CKLF-1 and other factors among the normal skin groups indicated that CKLF-1 may not show a standard high positive correlation with these inflammatory factors. Therefore, there might be a baseline of CKLF-1 expression required to activate the release of inflammatory factors.

As a novel human cytokine isolated from PHA-stimulated U937 cells,^6^ the study of CKLF-1's biological effects is still in the preliminary stage. Our study first assessed the expression level of CKLF-1 in keloid tissues in comparison with normal tissues and normal scar tissues, and it concluded that CKLF-1 expression and inflammation may be some of the causes of keloid. We also inferred that CKLF-1 may help identify keloid-predisposed individuals. It has been reported that CKLF-1 expression has a close relationship with the occurrence and development of inflammation. Thus, CKLF-1 expression may play an important role in the pathogenesis of inflammatory diseases and keloid formation. The mechanism of this process needs to be further explored.

CKLF-1 is overexpressed in keloid tissues, implying that CKLF-1 may have a functional role in keloid occurrence and development and that it may be a new target for the treatment of patients with keloid. Compared with normal skin tissues in patients without keloid, normal skin tissues in keloid patients also have a higher expression of CKLF-1; thus, CKLF-1 could be an indicating factor for keloid-predisposed individuals. To confirm the role in predisposition to keloids, more samples and in vitro studies and genetic researches are needed, which are the limitation of the study.

## CONCLUSION

In our study, CKLF-1 and other inflammatory factors were overexpressed in the samples from keloid patients, indicating that the formation of keloid may be related to inflammation and that CKLF-1 may play an important role in this process. In addition, there is significant difference of CKLF-1 expression in normal skin tissue between keloid and nonkeloid patients, implying that CKLF-1 may help predict keloids. In this case, CKLF-1 may become a new focus for researching mechanism of keloid formation.
